# Using Trauma Informed Principles in Health Communication: Improving Faith/Science/Clinical Collaboration to Address Addiction

**DOI:** 10.3389/fpsyg.2021.781484

**Published:** 2021-12-22

**Authors:** Andrea D. Clements, Natalie A. Cyphers, Deborah L. Whittaker, Bridget Hamilton, Brett McCarty

**Affiliations:** ^1^Department of Psychology, College of Arts and Sciences, East Tennessee State University, Johnson City, TN, United States; ^2^Uplift Appalachia, Johnson City, TN, United States; ^3^Ballad Health Strong BRAIN Institute, East Tennessee State University, Johnson City, TN, United States; ^4^Division of Nursing, DeSales University, Center Valley, PA, United States; ^5^Center for Nursing Research, College of Nursing, East Tennessee State University, Johnson City, TN, United States; ^6^Department of Population Health Sciences, School of Medicine, Duke University, Durham, NC, United States; ^7^Divinity School, Duke University, Durham, NC, United States

**Keywords:** health communication, faith-based health programming, trauma-informed approach, health information dissemination, faith and science, addiction, substance use and misuse

## Abstract

Problematic substance use is a pressing global health problem, and dissemination and implementation of accurate health information regarding prevention, treatment, and recovery are vital. In many nations, especially the US, many people are involved in religious groups or faith communities, and this offers a potential route to positively affect health through health information dissemination in communities that may have limited health resources. Health information related to addiction will be used as the backdrop issue for this discussion, but many health arenas could be substituted. This article evaluates the utility of commonly used health communication theories for communicating health information about addiction in religious settings and identifies their shortcomings. A lack of trusting, equally contributing, bidirectional collaboration among representatives of the clinical/scientific community and religious/faith communities in the development and dissemination of health information is identified as a potential impediment to effectiveness. The Substance Abuse and Mental Health Services Administration’s (SAMHSA) tenets of trauma-informed practice, although developed for one-on-one use with those who have experienced trauma or adversity, are presented as a much more broadly applicable framework to improve communication between groups such as organizations or communities. As an example, we focus on health communication within, with, and through religious groups and particularly within churches.

## Introduction

In cities and towns throughout the United States, people gather together in religious services. In fact, there are approximately 384,000 congregations of various denominations throughout the United States (Brauer, 2017). In Christian denominations alone, there are roughly 167 million people who may gather together in any given week (Pew Research Center, 2019). Within congregations, people attend for various purposes, some for worship, others to socialize, some for information sharing about their own lives as well as community, national, and global issues, and some because of a desire to enhance flourishing for themselves and others.

Since churchgoers are familiar with information sharing within their congregations, have a natural connection to their communities, and profess commitments to bodily health and flourishing, then disseminating health information to and through churches is sometimes seen as a valuable approach for reaching communities (Brewer and Williams, 2019; Idler et al., 2019). Such dissemination efforts have been on display during the COVID-19 pandemic through a variety of initiatives such as church-based vaccine clinics (Federal Emergency Management Association [FEMA], 2021) and efforts to persuade faith leaders to promote health information about COVID-19 (Abdul-Mutakabbir et al., 2021). There are many areas predating the pandemic in which the clinical/scientific community has sought to disseminate information through churches, such as in the arena of addiction prevention, treatment, and recovery (The Partnership Center, n.d.; Tennessee Department of Mental Health and Substance Abuse Services, 2021). Yet, these initiatives have met with limited success. The present models for health communication may be limited in their efficacy in these communities given their unidirectional approach *from* the clinical/scientific community *to* the faith community. Through an examination of the literature on health information dissemination in churches (Blevins et al., 2019) and the implicit communication theories which guide these practices, we propose an approach that may improve collaboration between the clinical/scientific community and the faith community, focusing on addiction while recognizing applications extend to many health conditions of interest.

Such collaboration would build off existing commitments and practices within faith communities. In the National Congregation Survey conducted in 2019, 33.2% of congregations reported having health-related programming within their churches (Chaves et al., 2020). Within these health programs there are variations in efficacy and structure. Two common and contrasting structures of health programs are faith-based and faith-placed approaches (DeHaven et al., 2004; Joseph et al., 2017; Baptiste-Roberts et al., 2021). Faith-based programs are health programs specifically designed with the faith communities’ values and beliefs in mind (DeHaven et al., 2004; Stewart, 2016; Johnston et al., 2018). Many faith-based programs have been developed to address various health concerns, such as described by Schwingel and Gálvez (2016) who implemented a behavioral life-style change program in a Latino church community that was taught by *promotoras*, church lay health advisors, who added bible readings and teachings relevant to the program. In contrast, faith-placed programs are health programs developed by outside sources and implemented “as is” (Stewart, 2016), for example, health screening implemented in churches (Lynch et al., 2020) or educational materials developed and implemented by health professionals in churches (Miller and Mars, 2020).

Faith-placed approaches, those without input from or modification to fit the faith community, continue to be used by health organizations to disseminate health information to and through churches, but are sometimes poorly received (Cochrane et al., 2014; Blevins et al., 2019; Tshiswaka et al., 2021). It is easy to see why such approaches are attractive to health organizations. They seem to offer widespread dissemination of carefully crafted and controlled health information by groups trusted in the local community. While some faith-placed strategies are effective, such as stroke education programs conducted within a church (Tshiswaka et al., 2021), health communication that is not informed by, or differs with, the church’s faith beliefs may be less effective (Blevins et al., 2019). Faith-placed strategies face certain limitations that faith-based programs, developed in collaboration with the communities they are intended to serve, do not. For example, faith-placed information, such as pamphlets, developed by health professionals may or may not be understood by the churchgoers, sometimes due to educational levels (Williams et al., 2019). Alternately, churchgoers may understand the material, but disagree on religious grounds or even be offended by the information at times as happened when the human papilloma virus (HPV) vaccine was first introduced (Touyz and Touyz, 2013). Lastly, churchgoers may not trust the source of the information due to previous experiences with an outside organization or general mistrust of the medical community (Jaiswal and Halkitis, 2019). In short, faith-placed approaches may be limited in their efficacy due to the limited emphasis placed on facilitating trust, ensuring congruence between materials and the churchgoers’ faith beliefs, and creating a process to ensure the members of the faith community understand the materials. There are topics on which it may be difficult or even impossible to align the views of particular faith communities and certain practices within the health sciences, from Jehovah’s Witnesses opposition to receiving blood transfusions (Crowe and DeSimone, 2019) to several Christian denominations opposing physician-assisted suicide (JW.org, 2021). These topics tend to amplify the chasm between science and faith and make communication challenging. However, these differences do not need to be seen as barriers to communication but opportunities to find common ground (Idler et al., 2019). When discussing potentially controversial topics, such as sex, the tenets of the faith community’s beliefs will impact how they receive information. For example, a faith community may feel that abstinence must be emphasized as a part of sex-education. For that reason, information about the HPV vaccine, HIV, STIs, and sex-education would need to non-judgmentally acknowledge that faith communities’ beliefs about abstinence as part of the framework in which the information is presented. Faith-based interventions should begin with identifying and recognizing the importance of these beliefs and the influence they may have on churchgoers in health decision-making. Without such acknowledgment of the faith community’s beliefs, health communication on these controversial topics will likely be compromised.

When discussing addiction treatment, clinical views often stand in some tension with those of many faith communities. For example, recent messaging around addiction seems designed to reject some efforts to make moral sense of substance use issues. The widely promoted phrasing, “Addiction is a chronic relapsing brain disease, NOT a moral failing” (paraphrased from multiple sources such as Leshner, 1997; Substance Abuse and Mental Health Services Administration [SAMHSA], 2018; MacKillop, 2020), sets up a dichotomy between solely physical views of addiction and perspectives that attempt to make room for moral agency and responsibility. While this phrase likely stems from well-intentioned and often-needed efforts to reduce stigma, such efforts do not need to assume that medical and moral/theological accounts of addiction are in conflict. These efforts to reduce stigma may find better success by aligning messages and enhancing collaboration. Many people of faith would say that both disease and moral agency must be considered in most issues related to health, including addiction (Rise and Halkjelsvik, 2019). Finding areas of alignment and collaboration begins with listening well and working to build trust in both directions.

Unfortunately, trust can be difficult to come by. The clinical community may believe that the church is not a reliable site for information dissemination or material distribution. They may see churchgoers as closeminded, uneducated, or uninformed. For example, in a qualitative study of 34 teams of faith community leaders and health community leaders, some community leaders noted that faith-based organizations “lack credibility” in disseminating health information (Kegler et al., 2010, p. 673). Some scientists believe that religious beliefs are not based in verifiable facts and therefore are less valuable than scientific information (Ecklund et al., 2011, 2016), perhaps leading to a less collaborative approach to dissemination of information in church settings. And while at times this skepticism may be warranted, its prominence in the medical community can prevent the recognition of possibilities for constructive collaboration. In 2019, a special issue section of the *American Journal of Public Health* (AJPH) explored faith/public health partnerships as a way to disseminate health information (Idler et al., 2019). By examining specific case studies and interventions, the articles and commentaries built a case for why faith communities should be taken seriously as potentially constructive collaborators. However, while the AJPH special issue sought to highlight the value that faith communities can play as vehicles for public health efforts, there was little attention to the bidirectional nature of full-fledged partnerships. Further focus on the give and take natural to trusting, robust collaborations is needed, and within that focus questions of the nature of health communication arise.

Recognizing the potential contributions religious communities might make in addressing public health crises and the need for bidirectional partnerships as part of such efforts, this article examines the health communication theories that often are at play within such collaborations. After analyzing why these approaches may have limited success in faith settings, we will propose a new approach to health communication that should enhance collaboration between clinical/scientific and faith communities, with an eye toward application to concerns surrounding addiction.

### Health Communication Theories

Utilization of effective health communication strategies has been touted as essential to the health outcomes of a community (Schillinger et al., 2020) including those related to addiction, yet theorists differ as to what is the best health communication strategy. We have chosen to present an overview of the two most commonly cited health communication theories, the Health Belief model (Rosenstock et al., 1988) and the Transtheoretical Model (Prochaska and Velicer, 1997). Then we will introduce a third model, Kleinman’s Theory of Explanatory Models (1978), which addresses some of the shortcomings of the others. Each of the theories will be explored specifically in relation to promoting health communication within churches. Finally, we will propose that a trauma-informed perspective offers a better perspective to guide health communication in partnership with faith communities.

#### The Health Belief Model

The Health Belief Model was developed in the early days of the United States Public Health Service in the 1950s to address ongoing issues with individual compliance to health interventions (Rosenstock et al., 1988). The model consists of four key constructs: perceived susceptibility, perceived severity, perceived benefits, and perceived barriers. These constructs are believed to influence an individual to engage in a behavior to prevent a health disease or condition (Rosenstock et al., 1988). In short, an individual must feel that they are susceptible to a disease or condition, that the disease or condition could be severe, that there are benefits to preventing the disease or condition, and that the benefits of health behavior change outweigh the barriers (Rosenstock et al., 1988). The theory posits that a *trigger* or cue to action, either internal (e.g., chest pains, shortness of breath) or external (e.g., advice of a family member or doctor), can motivate a new behavior due to the fact that a trigger will either increase or decrease perceived susceptibility, perceived severity, perceived benefits, or perceived barriers (Rosenstock et al., 1988). It is important to note that modifying factors such as demographics (e.g., age, education), sociopsychological factors (e.g., social class, personality), and structural factors (e.g., disease knowledge) also exert influence on health decision making.

When applying this theory, the clinical community seeks to initiate an external trigger to change behavior. This could be through sharing information about risky substance use or by identifying and addressing factors that may be barriers to change such as lack of knowledge about addiction treatment options (Rosenstock et al., 1988; Healthy People 2030 et al., 2021). According to Rosenstock et al. (1988), identification of the barrier should lead to education about the desired behavior and eventual behavior change. The strength of this model comes from its ability to focus on factors that may be preventing health behavior change (Rosenstock et al., 1988). However, critics argue that application of the model in practice is challenging due to the number of barriers that may influence the health behavior and choosing which is most significant to address (Jones et al., 2015). The Health Belief Model assumes stable health beliefs and tends to be provider focused. This deemphasis of the patients’ perspectives limits the understanding of their intention to perform a health behavior, which often involve motivators unrelated to health (Schwarzer, 2001).

Health organizations have drawn from the Health Belief Model in their efforts to disseminate health information in churches. They typically do so by choosing a health behavior and providing church-based education on that behavior (e.g., Martinez et al., 2016; White, 2018). This has often been in the form of *faith-placed* programming, which has the problems listed above, particularly the potential for distrust of the health messengers and possible incongruence between health materials and churchgoers’ faith beliefs. In these approaches, the attention to barriers described in the Health Belief Model generally narrows to a focus on education through one-way communication from the clinical community to the faith community. The cited deemphasis on the patient, or in this case, on the faith community, perspective, not only reduces the tailoring of the message to the audience, but misses a chance for collaborative trust building.

#### The Transtheoretical Model

Another well-known theory, the Transtheoretical Model (Prochaska and Velicer, 1997), attempts to remedy many of the stated criticisms of the Health Belief Model in ways that have important implications for engagements with faith communities. Rather than focusing on group-level dissemination of prepared health material, The Transtheoretical Model focuses more on individual-level change. It posits that individuals go through six stages (Precontemplation, Contemplation, Preparation, Action, Maintenance, and Termination) when choosing whether to change a health behavior (Prochaska and Velicer, 1997). Through discussion, a person is challenged by a trusted coach to move to the next stage through presentation of health material, under the assumption that the person will gradually be convinced to move toward behavior change in a stage-like fashion. This theory has yielded some success at achieving change in culturally diverse samples (Callaghan et al., 2005); however, critics state that the attempt to utilize stages does not consider the complexity of humans and human behavior (Adams and White, 2005; Brug et al., 2005).

Some of the success of the Transtheoretical Model is likely due to the dialogue and established trust between provider and patient and the autonomy of the patient to move at their own pace. However, though the patient has some autonomy over how quickly to move through the stages, the target of that movement is determined by the healthcare provider (e.g., smoking cessation is the goal, everyone should be vaccinated), thus is often provider driven and one-sided in content as was seen in the Health Belief Model. The stages of the Transtheoretical Model could be applied to work with an organization such as a church rather than with an individual; however, it still suffers from the criticism of one-way communication from the clinical/scientific community to the faith community.

Churches, which are often at the center of community life, present numerous opportunities for disseminating health information, thus it is important to find the best method for that dissemination. Both of these theories have been utilized frequently in public health interventions but suffer from limitations. They flow unidirectionally, limiting interaction between interventionists and the community itself. Buy-in from the community is essential in order to have an effective health intervention (Idler et al., 2019), thus an approach with more bidirectional input is indicated.

#### Kleinman’s Theory of Explanatory Models

Anthropologist and psychiatrist Kleinman (1978) sought to overcome unidirectional models of doctor patient interaction through the concept of the Theory of Explanatory Models. While this theory is focused on doctor-patient interactions, it could be used as a framework to inform communication between two communities (clinical/scientific and faith) rather than two individuals, and we will review it as such. Kleinman argued that both physicians and patients are influenced by culturally informed explanatory models which guide their understanding and treatment of illnesses. Kleinman recognized that patients’ explanatory models were influenced by personal, cultural, and social meaning ascribed to illness and recommended asking eight questions to elicit the patient’s explanatory model: 1. What do you call the problem? 2. What do you think has caused the problem? 3. Why do you think it started when it did? 4. What do you think the sickness does? How does it work? 5. How severe is the sickness? Will it have a long or a short course? 6. What kind of treatment do you think the patient should receive? 7. What are the chief problems the sickness has caused? 8. What do you fear most about the sickness? (McSweeney et al., 1997). Comparing the physicians’ and patients’ explanatory models was thought to illuminate discrepancies that existed and could then be discussed or “negotiated” with patients (Kleinman, 1978, p. 257). Understanding the differences between the patient and practitioner explanatory models provided a mechanism for conversations that, according to Kleinman, could attempt to “educate the patient” if the patient’s model was different than the physician’s (p. 257).

Since its inception, Kleinman’s theory has been expanded to look at both health and illness (McSweeney et al., 1997). The importance of negotiating with patients is emphasized rather than simply recommending educating patients about differences in health care providers’ and patients’ explanatory models (McSweeney et al., 1997; Kleinman and Benson, 2006). This negotiation should result in the patient feeling heard, thus valued, and should allow intervention to be better tailored to the specific situation. That value and tailoring should increase buy-in. Kleinman and Benson (2006) noted that explanatory models provide clinicians with the opportunity to walk alongside patients rather than elevating clinical/scientific understanding as superior. In a study by Daack-Hirsch and Gamboa (2010), Kleinman’s theory was used to describe the alignment of beliefs about cleft lip/cleft palate between healthcare workers and working people in the Philippines. Piven et al. (2008) studied the explanatory models about depression held by certified nursing assistants’ in nursing homes and compared their models to mood screening and diagnostic criteria for depression. In both of these studies, exploring explanatory models provided structure within which to consider how differences in illness beliefs may influence health communication between care providers and patients. Explanatory models are not static explanations within an entire culture, necessarily, rather they are changing and fluid because they include not only social and cultural beliefs, but also individuals’ and communities’ understanding of past experiences, knowledge, and their interplay (McSweeney et al., 1997). Kleinman and Benson (2006) noted explanatory models should be more like ethnography, truly emphasizing relationships and engagement with people to have the opportunity to hear their explanatory model and then moving forward together.

Kleinman’s theory does provide a communication theory that could facilitate dialogue between the clinical community and the faith community. By recognizing that churches have their own explanatory models with beliefs, norms, and values that may be very different from those of the clinical community, the need for collaboration when health information is disseminated within churches is paramount. In fact, each diverse religion, each religious group, each church, each member of each church, each branch of the clinical/scientific community, and each member of the clinical/scientific community will also have their own personal explanatory models. Such differences press the need for careful, charitable, and sustained dialogue.

Attention to articulating explanatory models and negotiating between those that differ gets us closer to the collaboration we believe is vital for accurate health information dissemination to and through faith communities, but in some cases, it still falls short. First, Kleinman’s theory tends to be focused on communication with an individual rather than a group, and we are proposing to equip large and diverse groups with valid health information, requiring an expanded focus beyond one on one communication. Second, although the patient’s beliefs are taken into consideration, it is unclear whether Kleinman provides for the possibility that the message itself could be altered by the perspective of the hearer, or just that the route of communicating the message would be altered. Often the healthcare provider still controls the goal of the message and simply seeks to understand how to deliver what is believed to be scientifically sound in a way that motivates the hearer to follow their guidance. In most instances we found, health *messaging* was modified by understanding the explanatory model of the faith community (Blevins et al., 2019), but the ultimate goal of that messaging usually was driven by the healthcare community. This is superior to messaging without consideration of the hearer’s explanatory model, but in the case of communication between the clinical/scientific community and the faith community, our hope is that collaboration will occur such that not only the delivery of the information is altered, but that perspectives of the faith community are incorporated into the message itself, as appropriate, within a genuine collaboration.

#### Trauma-Informed Perspective for Communication

We would like to propose a different approach to communication within churches involving the repurposing of what has come to be known as trauma-informed care, trauma-informed practice, or trauma-informed principles (Substance Abuse, and Mental Health Services Administration [SAMHSA], 2014). Trauma-informed principles were initially developed to guide one-on-one interactions between healthcare or social service providers and individuals who have experienced trauma, abuse, or adversity. This paradigm has not previously been applied to general health communication or to organization-level communication to our knowledge. We contend that trauma-informed principles, which we outline below, can and should be used much more broadly than originally intended. This breadth can include one-on-one use with anyone regardless of trauma history as well as more macro-level, organization to organization or community to community communication as we recommend here. We believe that trauma-informed principles address the shortcomings of prior health communication theories and may facilitate truly collaborative health messaging in faith communities. A trauma-informed perspective, like the Theory of Explanatory Models and the Transtheoretical Model, has been used as a way to facilitate communication between individuals and has also been used to create a culture within one organization to facilitate such interpersonal communication. We believe its tenets (e.g., empathy, open-mindedness, seeking to understand another’s perspective, not forcing one’s own agenda) can also be used at a more macro level to facilitate communication and collaboration between organizations and communities. It does so in a way that addresses several of the issues we noted above with the other three models.

We feel that it is important to introduce the origin of this theoretical framework, admitting the term trauma, though central to the theory’s origin, can be distracting and sometimes off putting. We ask the reader to refrain from focusing too closely on the term “trauma,” as we propose our broader view of this theory. A trauma-informed perspective is equated with viewing people, all people, through a lens of empathy, lack of judgment, and open-mindedly seeking their input into what they need (Substance Abuse, and Mental Health Services Administration [SAMHSA], 2014). The theory was initially developed in response to findings from the Adverse Childhood Experiences Study (ACEs) (Felitti et al., 1998; Substance Abuse, and Mental Health Services Administration [SAMHSA], 2014), emphasizing the importance of understanding that many people have experienced traumatic events, and that those experiences shape and explain poor behavior choices and ongoing health issues. Substance Abuse, and Mental Health Services Administration [SAMHSA], 2014 recommended what was then coined a *trauma-informed approach* as a type of universal precaution that included three E’s (i.e., Events can be traumatic, someone’s Experience of the event is most important, long-term Effects can be caused by the experience), four R’s (i.e., *Realize* widespread trauma, *Recognize* signs and symptoms indicating past trauma, *Respond* appropriately, *Resist* re-traumatization), and six principles. Those six principles of a trauma-informed approach will be presented as a framework for enhanced health communication within churches and are described in Table 1.

**TABLE 1:** SAMHSA’s six key principles of a trauma-informed approach applied to health communication.

Key principle	SAMHSA description focused on trauma*	Broad application for church/healthcare communication
Safety	Seek to ensure physical, emotional, and relational safety as defined by the person	Views can be expressed by all parties without fear of judgment. Collaborators are seen as allies.
Trustworthiness and transparency	Operations are conducted and decisions are made with transparency with the goal of building and maintaining trust	Clinical/scientific community and faith community members openly discuss views and seek to build trust through understanding each other’s perspectives. Domain specific knowledge is acknowledged.
Peer support	People with lived experience with adversity contribute to planning and provide mutual support	People with lived experience within the faith community and those with lived experience in the clinical/scientific community contribute to planning and provide mutual support
Collaboration and mutuality	Power differences are leveled and individuals work collaboratively	Members of the clinical/scientific community and faith community should work to place themselves and the other group on a level playing field. Acknowledge differing views, but do not let them become barriers. Find commonalities.
Empowerment, voice, and choice	Strengths should be capitalized on, individuals should be heard and helped to use their voices, and should be given choices, and those choices should be honored.	Members of the clinical/scientific community and the faith community should be heard and strengths of each point of view should be capitalized on. As health communication endeavors are developed, churches should have a voice in what is said and a choice in what to adopt.
Cultural, historical, and gender issues	It should be understood that a person’s culture, their own history, their culture’s history, and issues related to gender influence many things about them. This should not be written off or downplayed, but used as a way to better understand the person.	The clinical/scientific community should seek to understand the culture, history, and particular people and perspectives of the faith community, and the faith community should seek to understand the culture, history, and particular people and perspectives of the clinical/scientific community.

*Adapted from Substance Abuse, and Mental Health Services Administration [SAMHSA], 2014, (10–11).

While trauma-informed principles originated from research on interacting with individuals with trauma histories, we believe those tenets are applicable to communication much more generally and at the organization level. We propose that both fields involved in church-based health communication (e.g., church, clinical/scientific community) practicing the tenants of a trauma-informed perspective is the best way to enhance communication of, perceived value of, and dissemination of heath information in and through the church. Those tenets include empathizing with the other’s perspective, finding and utilizing strengths, and being collaborative and non-judgmental. Idler et al. (2019), in their introduction to an *American Journal of Public Health* special issue section regarding faith/public health collaboration, posited some best practices for communication between public health agencies and faith communities that nicely parallel trauma-informed practice. They included taking a ground-up, strengths identifying, listening approach with a goal of empowering stakeholders; respecting each organization’s domain expertise in collaborations; seeing faith leaders as allies (we expand this to recommend all individuals in one field see those in the other field as allies); recognizing ideological differences but not allowing them to become barriers to finding common goals; and maintaining long-term collaborations that can be activated when crises arise. Beyond these areas of overlap and resonance with Idler et al. (2019), trauma-informed principles would push even further to call for genuine empathetic dialogue and negotiation between clinical and faith communities.

Substance Abuse, and Mental Health Services Administration [SAMHSA] (2014) six principles appear to cover fairly isolated domains at first glance, however, when combined, can foster open, non-judgmental communication. How might this look in preparing health communication materials and programs for dissemination to and through the faith community? Individuals who are seeking to disseminate such materials should work with members of the faith community, seeking to understand their beliefs regarding the health behavior of interest in an open-minded, non-judgmental way. Trusted individuals who understand both the science and the tenets of the faith should be in such conversations, serving as liaisons between the groups. Facilitators may be needed to coach groups on the trauma-informed perspective to encourage open-mindedness, address power differentials, and offer reframing and rephrasing to prevent misunderstandings.

Ideally, members of the faith community and the clinical/scientific community will feel safe and heard, trust will be built between them, and messages and messaging can be developed through collaboration. The greatest hurdles we anticipate will be that some in the clinical/scientific community will be hesitant to open-mindedly explore the validity of faith perspectives that may stand in tension with commonly held public health perspectives, or they may struggle to understand that many faith communities both value health *and* prioritize other goods as well, rather than solely prioritizing the health and comfort of themselves or others. We do not propose that the clinical/scientific community must embrace or even believe all of the tenets of the faith community. Likewise, we do not expect the faith community to embrace all of the tenets of the clinical/scientific community. What we hope is that members of the faith community can be equipped with scientifically accurate knowledge and that scientifically accurate knowledge can be explored using the faith community’s frameworks of belief.

## Discussion

The kind of open-minded collaboration between the faith and clinical/scientific community we are suggesting must always be negotiated considering the particularities of these communities in each local environment. When this is done, health promoting messages and messaging can be created in ways that (1) align with the tenets of the faith, (2) are understandable to the faith community, and (3) that the faith community values. Such efforts will require commitment from individuals who are well versed in both clinical/scientific information *and* the tenets of the faith community through which materials are to be disseminated. To most effectively facilitate two-way communication, those bridge-builders with knowledge of clinical/scientific information and faith tenets and others involved in the health communication process need to be familiar with trauma-informed principles. This faith community alignment and buy-in should vastly improve health information dissemination.

Although our proposed ideas about enhancing health communication within churches is broadly applicable to many health conditions from COVID-19 vaccination to diabetes education, a trauma-informed approach to health communication related to addiction may be the impetus needed to begin to mobilize the faith community to partner with the clinical/scientific community to address the current large-scale addiction problem. So far, the efforts by either group alone to reduce deaths from addiction or lower the number of babies with neonatal abstinence syndrome have fallen short, but true collaboration in a respectful, equal partnership may start a movement that could turn the tide and change the world.

## Author Contributions

AC was central to formulating the direction of this article and the need for it, wrote most of the section on trauma-informed practice, and edited in light of her work in faith community mobilization to address addiction. NC was involved in initial framing of this article and wrote several drafts with much input from AC. DW was involved in initial framing of this article and contributed to and edited several early drafts with NC. BH gathered information on health communication theories and summarized them, read, and edited the health communication theory section. BM edited drafts of the original article, contributed several sources, and reframed content. He was instrumental in connecting theories and served as a theology expert. All authors contributed to the article and approved the submitted version.

## Conflict of Interest

The authors declare that the research was conducted in the absence of any commercial or financial relationships that could be construed as a potential conflict of interest.

## Publisher’s Note

All claims expressed in this article are solely those of the authors and do not necessarily represent those of their affiliated organizations, or those of the publisher, the editors and the reviewers. Any product that may be evaluated in this article, or claim that may be made by its manufacturer, is not guaranteed or endorsed by the publisher.
